# Gender differences in psychotropic medication prescribing in a rural area of Catalonia: a community-based observational study

**DOI:** 10.3389/fpubh.2026.1755070

**Published:** 2026-02-23

**Authors:** Elisabet Torrubia-Pérez, Anna Panisello-Tafalla, Silvia Reverté-Villarroya, Josep L. Clua-Espuny, David Piñol-Piñol, José Fernández-Sáez, Mireia Adell-Lleixà, Maria-Antonia Martorell-Poveda

**Affiliations:** 1 Nursing Department, Campus Terres de l'Ebre, Universitat Rovira i Virgili, Tortosa, Spain; 2 Advanced Nursing Research Group, Universitat Rovira i Virgili, Tarragona, Spain; 3 Primary Health-Care Center Tortosa Est, Institut Català de la Salut (ICS), Primary Care Service (SAP) Terres de l'Ebre, Tortosa, Spain; 4 Ebrictus Research Group, Research Support Unit Terres de l'Ebre, Institut Universitari d'Investigació en Atenció Primària Jordi Gol (IDIAP Jordi Gol), Tortosa, Spain; 5 Unitat de Suport a la Recerca Terres de l'Ebre, Fundació Institut Universitari per a la Recerca a l'Atenció Primària de Salut Jordi Gol i Gurina (IDIAPJGol), Tortosa, Spain; 6 Rehabilitation and Physical Medicine Department, Hospital de Tortosa Verge de la Cinta, Institut Català de la Salut (ICS), Tortosa, Spain; 7 Nursing Department, Campus Catalunya, Universitat Rovira i Virgili, Tarragona, Spain

**Keywords:** gender disparities, primary care, psychotropic medication prescribing, public health, rural health, women's mental health

## Abstract

**Introduction:**

Gender disparities in the diagnosis and treatment of common mental disorders are well documented, with women more frequently prescribed psychotropic medications than men. However, evidence from rural settings remains scarce. This study examined gender differences in psychotropic medication prescriptions among adults with mental health diagnoses in a rural region of Catalonia, Spain.

**Methods:**

A retrospective, population-based study was conducted using data from the primary care database. Adults with at least one diagnosis of anxiety, depression, mood disorder, sleep disorder, stress, or distress were included. Prescription patterns of psychotropic medications were analyzed by sex and age group using descriptive statistics, Chi-square tests, and multivariable logistic regression adjusted for age, number of prescribed medications, and diagnosis.

**Results:**

Among 32,748 individuals, 64.9% were women. Women were more frequently diagnosed with depression (27.4 vs. 22.5%), anxiety (57.1 vs. 53.5%), and mood disorders (4.2 vs. 2.8%; all *p* < 0.001), while sleep disorders were more common in men (34.9 vs. 32.3%, *p* < 0.001). Women were more likely to be prescribed anxiolytics (40.2 vs. 32.8%) and antidepressants (35.5 vs. 23.9%). Logistic regression confirmed higher odds of antidepressant (aOR = 1.52; 95% CI: 1.43–1.60) and anxiolytic use (aOR = 1.29; 95% CI: 1.22–1.36) among women, and lower odds for antipsychotics (aOR = 0.68; 95% CI: 0.61–0.76). Disparities were most pronounced among women aged 45–64 years.

**Conclusion:**

Women in rural Catalonia were more often diagnosed with mental disorders and prescribed psychotropic medications, particularly antidepressants and anxiolytics. A gender-sensitive approach in primary care, including expanded access to psychological therapies and community-based services, is needed to address inequities.

## 1 Introduction

Mental health constitutes an increasing global challenge, with a high prevalence of disorders such as depression and anxiety, as well as other common problems such as stress or psychological distress (1). Although several studies conducted in rural areas suggest that these environments may exert a protective effect against common mental disorders (2), other research contradicts this by indicating that limited access to mental health services and other healthcare inequalities may lead to inadequate management (3).

The medicalisation of these common mental disorders shows clear gender differences. Psychotropic medications are substances that act on the brain to influence mood, perception, cognition, or behavior, and are used to treat mental health disorders (4). Women are prescribed psychotropic medications—particularly antidepressants and anxiolytics—more frequently than men across various clinical and population contexts, even after adjusting for sociodemographic and psychiatric diagnostic factors. For instance, the likelihood of antidepressant prescription is consistently higher among women, both in primary care and psychiatric settings, and this pattern has been observed in multiple European and Latin American countries (5–8).

In Spain, a recent study found that women had a 1.74-fold higher probability of being diagnosed with common mental disorders and a 1.26-fold higher likelihood of psychotropic medication consumption compared with men (9). This may be partially attributed to gender-related factors, such as the historically unequal development of mental health problems or the differential use of healthcare services; however, these factors alone do not fully explain the pattern. A study in the Basque Country revealed that women were 2.48 times more likely to receive diagnoses of depression or anxiety and, even after adjusting for symptoms and medical visits, still exhibited a 52% higher rate of psychotropic drug use (10). In rural Spanish communities, women's consumption of psychotropic medication more than doubled that of men, with 10.7% of women vs. 2.6% of men reporting use (11), although recent literature on these non-urban environments remains scarce.

When analyzing age group differences, in adolescence, boys receive psychotropic medications more frequently than girls, but this pattern reverses in young adulthood, when women exhibit significantly higher prescription rates, particularly of antidepressants and anxiolytics. The gap widens among women aged 45–54 years—coinciding with the menopausal transition—when the incidence of depression and anxiety diagnoses, as well as antidepressant prescriptions, is notably higher than among men of the same age. In older adults, the gender gap tends to persist, though it may slightly decrease at more advanced ages (6, 12).

At the same time, current trends point to a sustained increase in the use of these medication, especially among women and following the COVID-19 pandemic. A study conducted in northern Spain in 2021 found that 372 per 1,000 women took psychotropic medication daily, compared with 184 men, and the post-pandemic increase was significant in both sexes (13). On the other hand, studies conducted in rural settings remain scarce, despite their distinct characteristics: aging populations, limited access to specialized services, and greater geographical dispersion, which restrict non-pharmacological alternatives such as psychological therapy and foster increased pharmacological prescriptions. A study in a rural Spanish community found that 10.7% of women used psychotropic medications compared with 2.6% of men, with consumption strongly associated with physical comorbidity (14).

The literature indicates that in rural areas, prescription patterns are influenced both by access to mental health services and by sociodemographic and gender-related factors. Among older adults with severe mental illnesses, it has been documented that primary care physicians in rural areas prescribe most psychotropic medications. Although studies do not always explicitly report sex-based differences in prescribing practices, the greater prescribing burden in primary care in rural vs. urban areas may amplify gender differences observed elsewhere (15). Moreover, it has been noted that women in rural environments and small urban centers use mental health services and psychotropic medication more frequently than men, and that resource limitations have a greater impact on women's use of services and prescription patterns than on men's (16).

These findings suggest that gender differences in psychotropic medication prescribing persist and may even be accentuated in rural contexts, particularly among women exposed to higher social vulnerability. However, the available literature highlights the need for further studies explicitly examining sex differences in prescribing practices in these settings (15, 16). The potential influence of access to psychological therapies in rural areas is addressed in the Discussion.

In this context, Terres de l'Ebre—with a population density of 54.9 inhabitants/km^2^ in 2021 (17) and notable geographical dispersion—constitutes a non-urban rural region that provides a strategic setting for analyzing these phenomena from a gender perspective. The lack of infrastructure, distance from mental health centers, population aging, and limited access to specialized services could exacerbate both existing inequalities and potential clinical biases. Thus, the present study aims to assess gender differences in pharmacological prescription among men and women diagnosed with mental health problems in a rural area.

## 2 Material and methods

### 2.1 Study design and data source

A retrospective, descriptive, population-based observational study was conducted using clinical data extracted from the SIDIAP database (Information System for Research in Primary Care), a management tool for electronic medical records used by primary care centers within the Catalan Institute of Health (ICS, for its Catalan acronym). The ICS provides publicly funded primary healthcare coverage to the majority of the population in Catalonia. In the Terres de l'Ebre Health region, primary care services delivered by the ICS constitute the main point of access to healthcare for the population, ensuring broad population coverage and longitudinal follow-up. Therefore, the SIDIAP database is considered a reliable source for population-based epidemiological research in this setting. Data extraction was performed in March 2021, retrieving all available information covering the previous 10 years. The study followed the STROBE guidelines for observational research.

### 2.2 Study population

The study population consisted of men and women residing in the Terres de l'Ebre Health Region, a rural area located in southern Catalonia (Spain). According to the 2021 demographic report, this region has 180,794 inhabitants, of whom 76,432 are women and 77,623 are adult men (18). Given the public and universal nature of the primary care system in this region, the majority of adult residents have an active medical record within the ICS network, supporting the representativeness of the source population captured in the SIDIAP database.

### 2.3 Inclusion and exclusion criteria

The sample was obtained through intentional non-probabilistic sampling, applying predefined inclusion and exclusion criteria applied to the entire adult population registered in the regional primary care system. Inclusion criteria were: (1) aged 18 years or older, (2) having an active medical record, (3) being registered in the Terres de l'Ebre Health Region, and (4) having at least one of the following diagnoses recorded in their primary care medical history: anxiety, depression, sleep disorder, persistent mood disorder, stress, and distress. Exclusion criteria included habitual residence outside the region at the time of inclusion or diagnoses made prior to 2010. After applying these criteria to the total regional population (*N* = 180,794) (18), the final database comprised 32,748 cases.

### 2.4 Study variables

Variable definitions were based on those available in the SIDIAP database. Sex was considered the independent variable and was recorded only in binary form (male–female), as registered in the electronic health records. Information on gender as a social construct was not available in the database. However, the analysis was conducted within a gender perspective framework, acknowledging that gender-related social roles, norms, and expectations may influence mental health diagnosis and psychotropic prescribing. Regarding dependent variables, age was categorized into four groups−18–24, 25–44, 45–64, and ≥65 years—according to the classification of the National Statistics Institute for health determinant analysis (19). Diagnoses were categorized according to the *International Classification of Diseases ICD-10-ES* (20). As diagnoses were based on clinician-assigned codes, some degree of misclassification cannot be ruled out, both in the diagnoses and in possible gender biases. The diagnostic categories included anxiety, depression, sleep disorders, persistent mood disorders, stress, and distress. Although categories such as stress and distress are less specific from a nosological perspective, they were retained in the analysis to reflect real-world clinical practice and to capture the full spectrum of common mental health-related presentations managed in primary care settings.

Psychotropic medications were grouped according to pharmacological families defined by the globally accepted classification system (21). Besides, the count of psychotropic medication prescribings reflected the number of different psychotropic medications prescribed to each individual at the specific cross-sectional time point at which study data were extracted. Thus, the count represents the number of distinct psychotropic medications concurrently prescribed at that moment, rather than cumulative prescriptions over time.

To analyse psychotropic prescription volume by age and gender, the variables “no medication”, “1–4 medications prescribed”, and “≥5 medications prescribed” were created. The first included individuals who, despite having one or more diagnoses, had not yet been prescribed psychiatric medication, whereas the latter referred to those considered polypharmacologically treated with psychotropic medications (≥5 prescriptions). The threshold of five or more concurrently prescribed psychotropic medications was used as the operational definition of psychotropic polypharmacy, in accordance with commonly used descriptive criteria in pharmacoepidemiological research and clinical practice. The category reflects a high level of concurrent psychotropic treatment at the time of data collection.

### 2.5 Statistical analysis

Data were analyzed using IBM SPSS Statistics for Windows (Version 27.0). Descriptive statistics were used to detail the sociodemographic characteristics of the sample and the volume of prescribed medication. Normality of quantitative variables was assessed, and for categorical variables, dependency relationships were examined using the Chi-square test to explore bivariate associations between sex and dependent variables.

Finally, pharmacological families were incorporated into a logistic regression model, and adjusted odds ratios (aOR) by age, number of prescribed medications, and diagnosis were calculated to determine the strength of the association between prescribed pharmacological classes and sex. The significance level was set at *p* < 0.05.

### 2.6 Ethical consideration

This study was approved by the Clinical Research Ethics Committee of the *Institut Universitari d'Investigació en Atenció Primària Jordi Gol* (*IDIAP Jordi Gol*) under code 20/157-P in December 2020. Data extraction was carried out in a fully anonymised manner, and the information obtained was used solely for scientific purposes.

## 3 Results

### 3.1 Sample characteristics

After applying the inclusion and exclusion criteria, the final sample consisted of 32,748 subjects, of whom 64.9% were women (*n* = 21,262) and 35.1% were men (*n* = 11,486). Table 1 shows sex differences across age groups: men were more prevalent in the 18–24 and 25–44 age groups (*p* < 0.001 in both cases), whereas women predominated in the ≥65 age group (*p* < 0.001).

**Table 1:** Sociodemographic characteristics of the participants.

**Variable/Items**	Total		Women		Men		
	***n***	**%**	***n***	**%**	***n***	**%**	***p***
Total	32,748		21,262		11,486		
**Age**							
18–24	1,261	3.9	750	3.5	511	4.4	< 0.001^*^
25–44	7,299	22.3	4,424	20.8	2,875	25.0	< 0.001^*^
45–64	11,804	36.0	7,618	35.8	4,186	36.4	0.269
More than 65	12,384	37.8	8,470	39.8	3,914	34.1	< 0.001^*^
**Mental health diagnoses**							
Depression	8,425	25.7	5,836	27.4	2,589	22.5	< 0.001^*^
Persistent mood disorder	1,213	3.7	896	4.2	317	2.8	< 0.001^*^
Anxiety	18,294	55.9	12,151	57.1	6,143	53.5	< 0.001^*^
Stress	1,943	5.9	1,295	6.1	648	5.6	0.101
Sleep disorder	10,891	33.3	6,878	32.3	4,013	34.9	< 0.001^*^
Distress	1,848	5.6	1,344	6.3	504	4.4	< 0.001^*^
**Number of mental health diagnoses**							
1	24,384	74.5	15,260	71.8	9,124	79.4	< 0.001^*^
2	7,020	21.4	4,984	23.4	2,036	17.7	< 0.001^*^
3	1,344	4.1	1,018	4.8	326	2.8	< 0.001^*^
**Prescribed medication**							
Anxiolytics	12,318	37.6	8,554	40.2	3,764	32.8	< 0.001^*^
Antidepressants	10,282	31.4	7,542	35.5	2,740	23.9	< 0.001^*^
Mood stabilizers	3,064	9.4	2,045	9.6	1,019	8.9	0.027^*^
Antipsychotics	1,635	5.0	982	4.6	653	5.7	< 0.001^*^
Psycholeptics	21	0.1	18	0.1	3	0.0	0.046^*^

Chi-Squared test.

^*^Significant at *p* < 0.05.

Regarding mental health diagnoses, anxiety was the most frequent condition (55.9%), followed by depression (25.7%) and sleep disorders (33.3%). Women exhibited higher prevalence rates of depression (27.4 vs. 22.5%), mood disorders (4.2 vs. 2.8%), anxiety (57.1 vs. 53.5%), and distress (6.3 vs. 4.4%) compared with men (*p* < 0.001 in all cases). Conversely, sleep disorders were more frequent among men (34.9 vs. 32.3%, *p* < 0.001). Moreover, men were more likely to present a single diagnosis (79.4 vs. 71.8%), while women more often had two or three diagnoses (*p* < 0.001).

Concerning pharmacological prescription, anxiolytics (37.6%) and antidepressants (31.4%) were the most frequently prescribed medication classes. Women received anxiolytics (40.2 vs. 32.8%) and antidepressants (35.5 vs. 23.9%) more often than men (*p* < 0.001 in both cases). Differences were also statistically significant for antiepileptics (*p* = 0.027) and antipsychotics (*p* < 0.001), although the magnitude of these differences was smaller.

### 3.2 Prescribed psychotropic medications by sex and age group

Analysis by number of prescribed medications showed that men were more likely not to receive any psychotropic medication in all age groups (*p* < 0.001), whereas women aged 45–64 and ≥65 years had higher rates of prescription for 1–4 psychotropic medications (*p* < 0.001). No significant sex differences were observed in polypharmacy (≥5 medications; Table 2).

**Table 2:** Number of psychotropic medications prescribed by sex and age group.

**Number of medication prescribed**	**Age**	**Total**	Women		Men		***p***
			***n***	**%**	***n***	**%**	
		32,748	21,262		11,486		
No medication	18–24	1,049	625	2.9	424	3.7	< 0.001^*^
	25–44	5,126	3,030	14.3	2,096	18.2	< 0.001^*^
	45–64	5,895	3,556	16.7	2,339	20.4	< 0.001^*^
	More than 65	3,004	1,865	8.8	1,139	9.9	0.001^*^
1–4 medications prescribed	18–24	210	124	0.6	86	0.7	0.073
	25–44	2,115	1,360	6.4	755	6.6	0.534
	45–64	5,765	3,976	18.7	1,789	15.6	< 0.001^*^
	More than 65	9,261	6,519	30.7	2,742	23.9	< 0.001^*^
≥5 medications prescribed	18–24	2	1	0.0	1	0.0	0.658
	25–44	58	34	0.2	24	0.2	0.314
	45–64	144	86	0.4	58	0.5	0.190
	More than 65	119	86	0.4	33	0.3	0.093

Chi-Squared test.

^*^Significant at *p* < 0.05.

**Figure 1:**
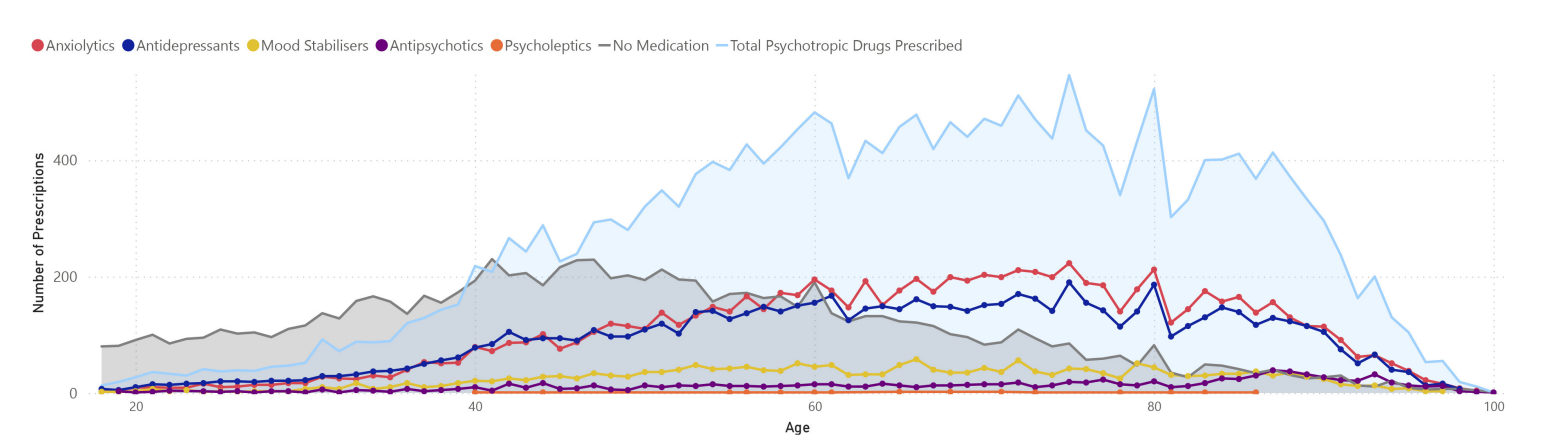
Prescribed psychotropic medications among women diagnosed with common mental health disorders.

**Figure 2:**
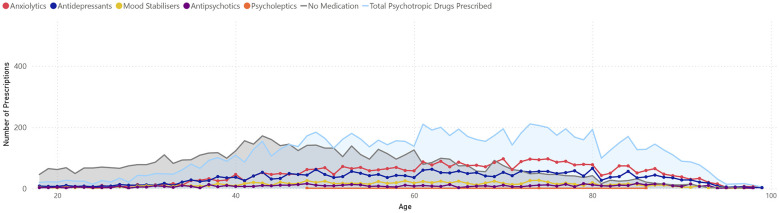
Prescribed psychotropic medications among men diagnosed with common mental health disorders.

Figures 1, 2 show the age distribution of prescribed psychotropic medications among women and men with mental health disorders. In women, the total number of prescribed medications gradually increases from age 20, peaking roughly between 65 and 75 years, and then declining in older ages. Among the medication categories, anxiolytics and antidepressants are the most frequently prescribed, whereas mood stabilizers, antipsychotics, and psycholeptics are less common. The proportion of individuals without medication is relatively high at younger ages and decreases toward the peak of prescriptions.

In men, the overall trend is similar, although the total number of prescribed medications is slightly lower than in women across most age ranges. Anxiolytics and antidepressants also predominate, with lower peaks compared to women. The proportion of men without prescriptions follows a comparable pattern, higher at younger ages and decreasing through middle age.

### 3.3 Logistic regression analysis

The logistic regression model adjusted for age and diagnosis showed significant differences in psychotropic medication prescribing according to sex. Compared with men, women had a higher likelihood of receiving anxiolytics (aOR = 1.29; 95% CI: 1.22–1.36; *p* < 0.001) and antidepressants (aOR = 1.52; 95% CI: 1.43–1.60; *p* < 0.001). No significant differences were observed in the prescription of mood stabilizers (aOR = 0.99; 95% CI: 0.91–1.07; *p* = 0.758; Table 3).

**Table 3:** Logistic regression model of prescribed psychotropic medications, adjusted for age, number of prescribed medications, and diagnoses.

**Pharmacological family**	**Sex**	**aOR^a^**	95% CI		***P***
Anxiolytics	Men	1			
	Women	1.29	1.22	1.36	< 0.001^*^
Antidepressants	Men	1			
	Women	1.52	1.43	1.60	< 0.001^*^
Mood stabilizers	Men	1			
	Women	0.99	0.91	1.07	0.758
Antipsychotics	Men	1			
	Women	0.68	0.61	0.76	< 0.001^*^
Psycholeptics	Men	1			
	Women	2.7	0.79	9.24	0.114

^a^Adjusted odds ratio.

^*^Significant at p < 0.05.

Conversely, women had a lower probability of receiving antipsychotics (aOR = 0.68; 95% CI: 0.61–0.76; *p* < 0.001). In the case of psycholeptics, although the adjusted odds ratio was higher (aOR = 2.70), the association did not reach statistical significance (95% CI: 0.79–9.24; *p* = 0.114).

## 4 Discussion

This study analyzed gender differences in the prescription of psychotropic medications among adults diagnosed with common mental disorders in a rural area of Catalonia. The findings revealed that women were more frequently diagnosed with depression, anxiety, and mood disorders, and were prescribed anxiolytics and antidepressants at significantly higher rates compared to men. In contrast, men were more likely to remain without pharmacological treatment despite a documented mental health diagnosis, and had slightly higher rates of sleep disorder diagnoses and antipsychotic prescriptions. However, the absence of information on symptom severity and non-pharmacological treatments limits the ability to determine whether these prescribing differences reflect clinical complexity, treatment availability, or differences in prescribing practices.

Our findings are consistent with prior studies in Spain and other European countries, which have repeatedly shown higher psychotropic use among women (7, 9, 10, 22), even after adjusting for symptoms and health service use. This pattern raises concerns about the potential medicalization of women's mental health. As the observed differences cannot be fully explained by clinical need alone, they may reflect underlying systemic gender biases in diagnostic practices and prescribing, with women being more readily labeled and treated pharmacologically. Socially constructed factors—such as gendered expectations, health-seeking behaviors, and provider decision-making—may reinforce these disparities, underscoring the potential over-pathologization and excessive pharmacological treatment of women's psychological distress.

People are exposed to social determinants across their life course that were shaped by systems and institutions of power, which often produce and reproduce systemic gender inequities (23–26).

### 4.1 Influence of the rural context

The rural context of Terres de l'Ebre in South Catalonia provides an additional layer of complexity (27). Similar to other rural areas, structural limitations such as fewer specialized mental health services, longer distances to care, and a higher prevalence of comorbid chronic conditions may foster greater reliance on pharmacological treatments, particularly among women. International evidence indicates that primary care physicians in rural areas are the main prescribers of psychotropic medications, with higher prescribing rates than in urban areas and greater responsibility due to a scarcity of non-pharmacological treatment options like psychotherapy and specialists, such as psychiatrists and behavioral health specialists. This situation places significant clinical and ethical burdens on the management of complex mental health needs (15, 28). The potential influence of limited access to psychological therapies should therefore be interpreted as a contextual factor warranting further investigation. The greater use of psychotropic medications among women may point to a dual vulnerability: an increased burden of mental health disorders alongside more limited access to non-pharmacological interventions. Structural inequities—such as the scarcity of psychological services in rural areas and the lack of gender-sensitive approaches in mental healthcare—may exacerbate this reliance on medications, reinforcing the medicalization of women's psychological distress and perpetuating gender-based disparities in treatment (10, 29). As this study does not include an urban comparison group, these findings should be interpreted as context-specific to the rural setting studied.

### 4.2 Gender and age-specific patterns and their implications for clinical practice and policy

Interestingly, the analysis by age group showed that gender disparities were most pronounced in middle-aged and older women, particularly between 45 and 64 years, a period that coincides with the menopausal transition. This finding is consistent with recent literature linking hormonal changes to an increased risk of depressive and anxiety symptoms, and to higher prescription rates of antidepressants during this life stage (23), although this association cannot be directly examined in the present study and should therefore be interpreted as a contextual hypothesis rather than a causal explanation.

The implications of these findings are relevant for both practice and policy (30, 31). From a clinical perspective, the results may have direct implications for incorporating a gender-sensitive approach in the assessment and management of mental health conditions, especially in rural primary care. Men and women often experience mental illness differently and seek care in distinct ways. Strengthening community-based mental health services and adopting a gender-sensitive approach make services more accessible and effective by addressing these gender-based disparities, promoting open communication, tailoring treatments to individual needs, and equipping providers with the skills to understand and respond to diverse gender-specific challenges by designing effective prevention strategies that intervene on modifiable social risk factors.

From a broader policy perspective, and beyond what can be directly inferred from the present data, system-level interventions should be regarded as priorities for future research and policy development, rather than as conclusions directly supported by this study. Further research is also needed to examine how the characteristics of health professionals—such as gender, training, and years of experience—may influence prescribing practices, as well as to assess the impact of geographic barriers on the accessibility and use of alternative mental health resources.

### 4.3 Strengths and limitations

This study has several limitations. First, the use of routinely collected clinical data may lead to underreporting or misclassification of diagnoses. In addition, information on relevant potential cofounders—such as comorbid physical conditions or socioeconomic determinants—were not available in the database and therefore could not be included in the adjusted analyses. Second, although the analysis adopts a gender perspective, only binary sex was available in the dataset, which restricts a more nuanced analysis of diversity. Therefore, gender-related influences could not be directly measured and are inferred indirectly. As such, interpretations related to gender differences should be understood as contextual and hypothesis-generating rather than as direct measures of gender effects. Third, the possibility of indication bias or recording bias inherent in administrative data. Finally, the observational design precludes establishing causal relationships between gender and prescription patterns. While biological factors contribute to higher rates of certain mental health issues in women (32), it is also possible that this disparity reflects over-medicalization in women and under-treatment in men, highlighting a need for deeper investigation into the complex interplay of factors contributing to gender differences in mental health treatment. Nonetheless, the large population-based sample and the focus on a rural area where evidence remains scarce strengthen the contribution of this work. The manuscript addresses an important and timely topic concerning gender disparities in mental healthcare from non-urban populations in Spain and the rural Catalan region, a setting often underrepresented in the literature, which enhances the originality and relevance of the study. The integration of a gender-sensitive framework and the focus on primary care practice make the contribution valuable to the field of public and rural health research.

### 4.4 Future directions

This study demonstrates that gender differences in psychotropic medication prescribing persist in rural Catalonia, with women being more frequently diagnosed with mental health conditions and more often prescribed anxiolytics and antidepressants than men. These disparities are shaped not only by clinical needs but also by gendered patterns of healthcare utilization and by structural constraints in rural health systems. In this context, the findings highlight the importance of ongoing education and sensitization of primary care health professionals regarding psychotropic medication prescribing. Future research should further explore the intersection between gender, social vulnerability, and access to non-pharmacological mental health interventions in rural populations. Furthermore, it would be important to investigate how the characteristics of health professionals—such as gender, training, and years of experience—influence prescribing practices, as well as to assess the impact of geographic barriers on the accessibility and use of alternative mental health resources.

A gender-sensitive approach in rural primary care is needed to address the dual risk of over-medicalization in women and under-treatment in men, while broader structural interventions should be evaluated through dedicated policy-oriented and comparative research designs. Further research should examine how expanding access to non-pharmacological interventions—such as community-based mental health services, gender-sensitive training for primary care providers, and psychotherapy—might contribute to reducing inequities,

## 5 Conclusions

In this rural population, women were more likely to be diagnosed with common mental disorders and prescribed psychotropic medications, particularly antidepressants and anxiolytics. These findings reflect not only clinical needs but also potential gendered patterns in healthcare utilization and prescribing.

## Data Availability

The raw data supporting the conclusions of this article will be made available by the authors, without undue reservation.
